# Supplementary figures and images for: Caspase-mediated nuclear pore complex trimming in cell differentiation and endoplasmic reticulum stress
Source: eLife. 2023 Sep 4;12:RP89066. doi: 10.7554/eLife.89066 (PMC10476967; doi:10.7554/eLife.89066)

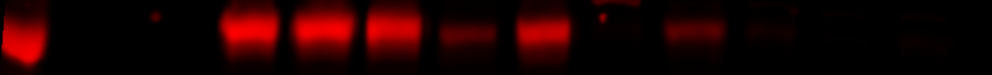

Supplement: Figure 1—source data 1. [file elife-89066-fig1-data1.zip › Figure 1 - source data 1/Figure 1g - source data/2. Zyxin.tif]

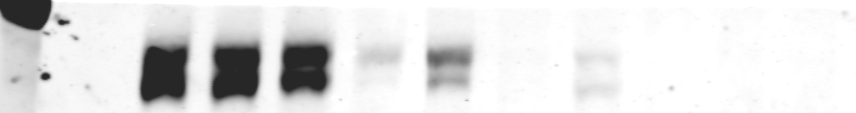

Supplement: Figure 1—source data 1. [file elife-89066-fig1-data1.zip › Figure 1 - source data 1/Figure 1g - source data/3. Paxillin.tif]

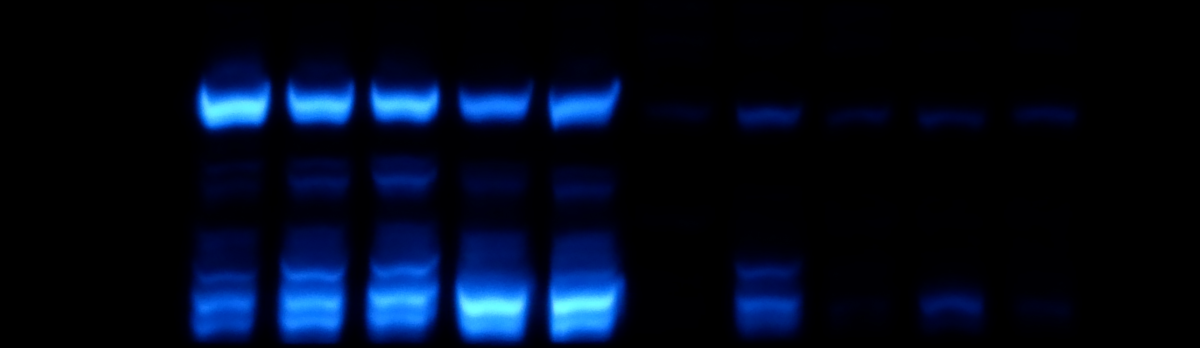

Supplement: Figure 1—source data 1. [file elife-89066-fig1-data1.zip › Figure 1 - source data 1/Figure 1g - source data/4. FAK.tif]

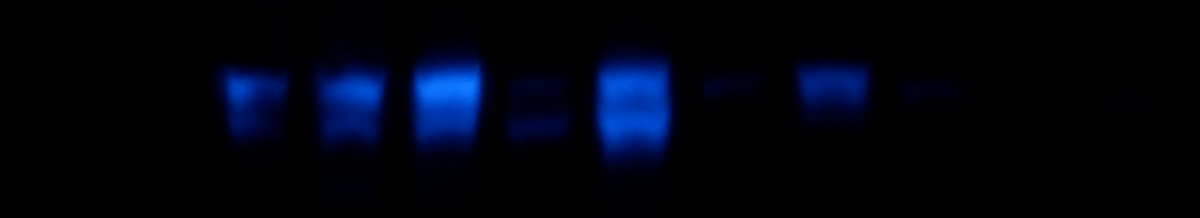

Supplement: Figure 1—source data 1. [file elife-89066-fig1-data1.zip › Figure 1 - source data 1/Figure 1g - source data/1. Hic-5.tif]

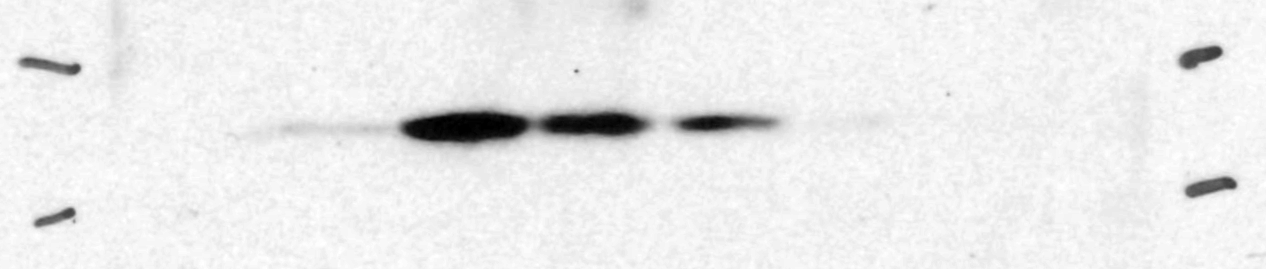

Supplement: Figure 1—source data 1. [file elife-89066-fig1-data1.zip › Figure 1 - source data 1/Figure 1a - source data/8. casp3.tiff]

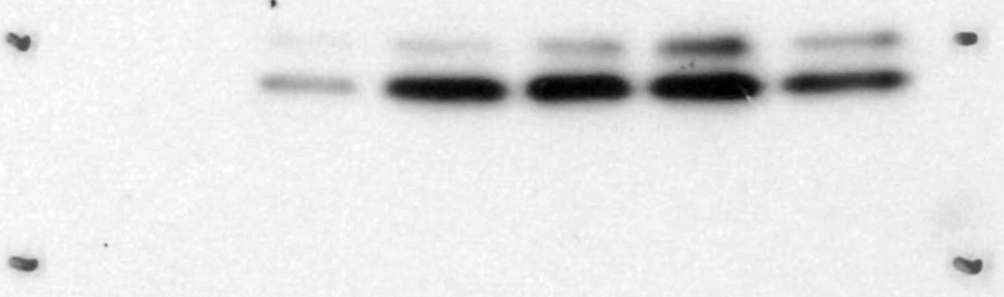

Supplement: Figure 1—source data 1. [file elife-89066-fig1-data1.zip › Figure 1 - source data 1/Figure 1a - source data/9. Myog.tiff]

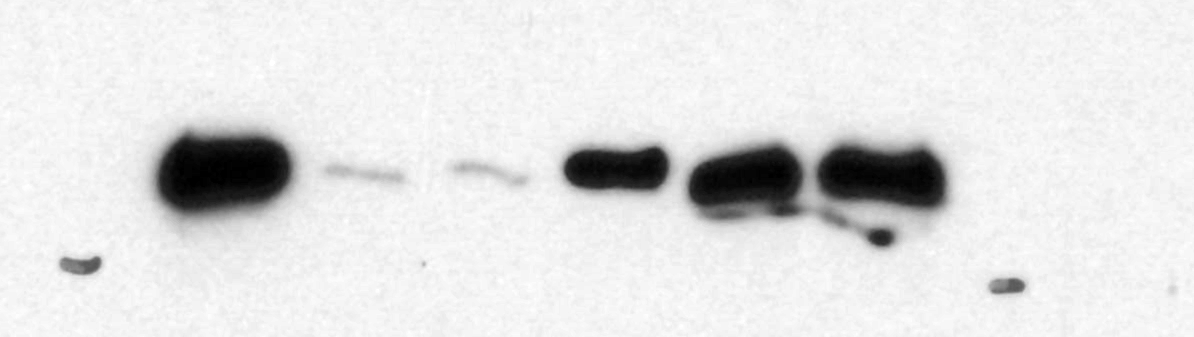

Supplement: Figure 1—source data 1. [file elife-89066-fig1-data1.zip › Figure 1 - source data 1/Figure 1a - source data/3. Tpr.tiff]

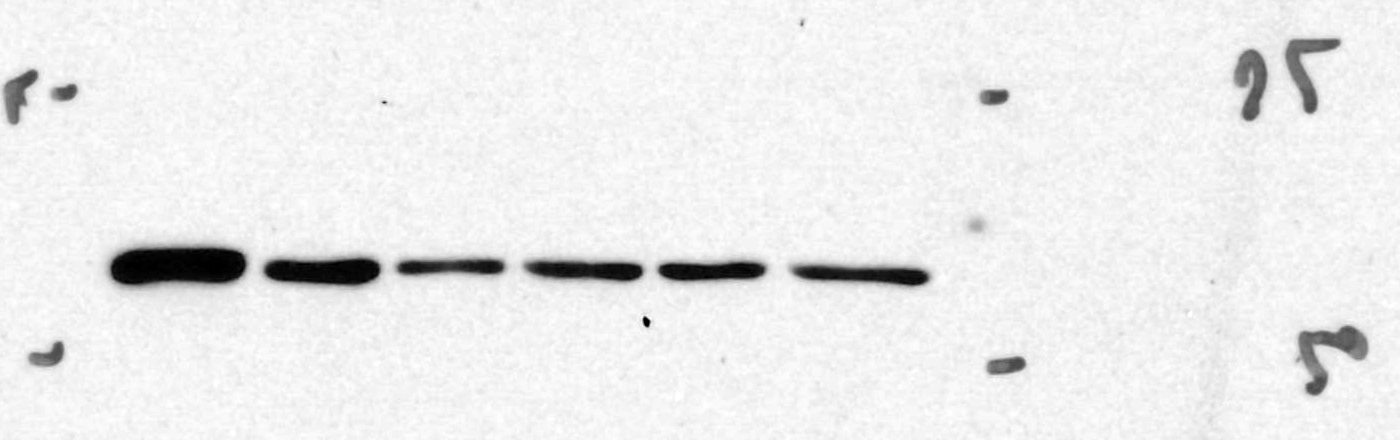

Supplement: Figure 1—source data 1. [file elife-89066-fig1-data1.zip › Figure 1 - source data 1/Figure 1a - source data/6. Nup50.tiff]

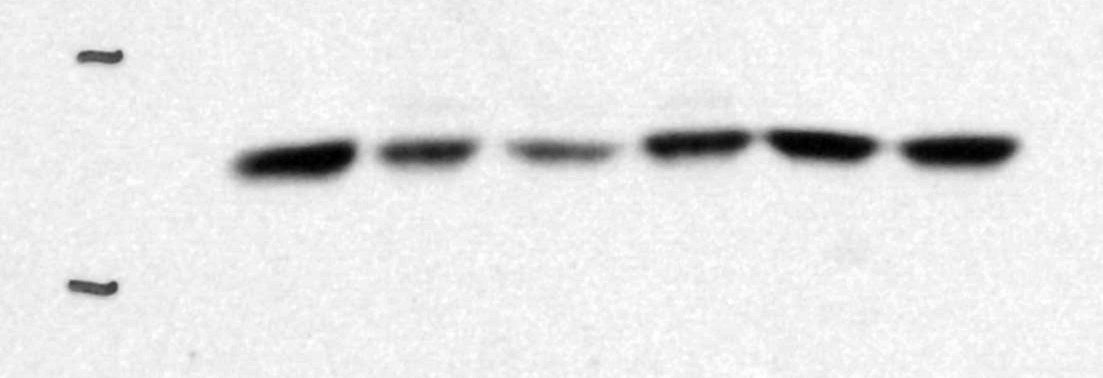

Supplement: Figure 1—source data 1. [file elife-89066-fig1-data1.zip › Figure 1 - source data 1/Figure 1a - source data/2. Nup62.tiff]

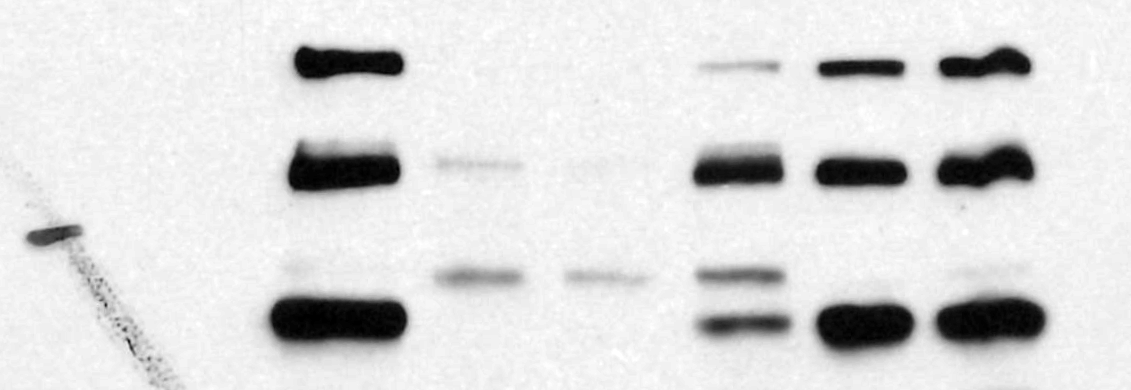

Supplement: Figure 1—source data 1. [file elife-89066-fig1-data1.zip › Figure 1 - source data 1/Figure 1a - source data/1. Nup358 214 153.tiff]

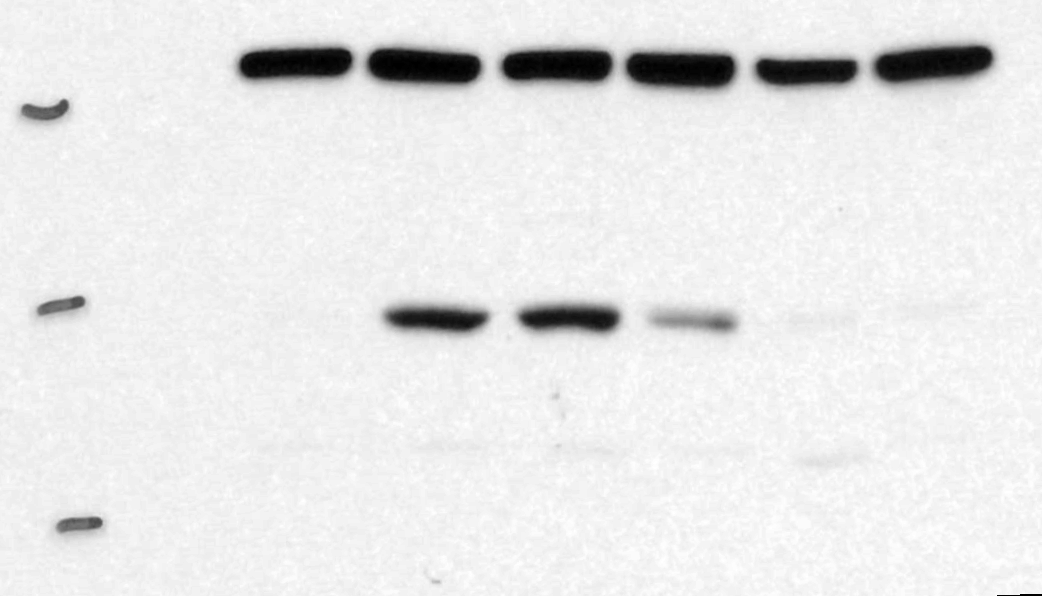

Supplement: Figure 1—source data 1. [file elife-89066-fig1-data1.zip › Figure 1 - source data 1/Figure 1a - source data/11. aII-spectrin.tiff]

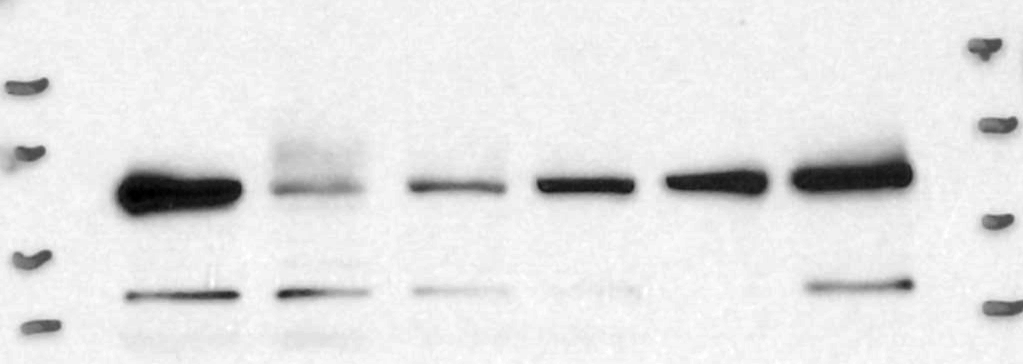

Supplement: Figure 1—source data 1. [file elife-89066-fig1-data1.zip › Figure 1 - source data 1/Figure 1a - source data/10. PARP.tiff]

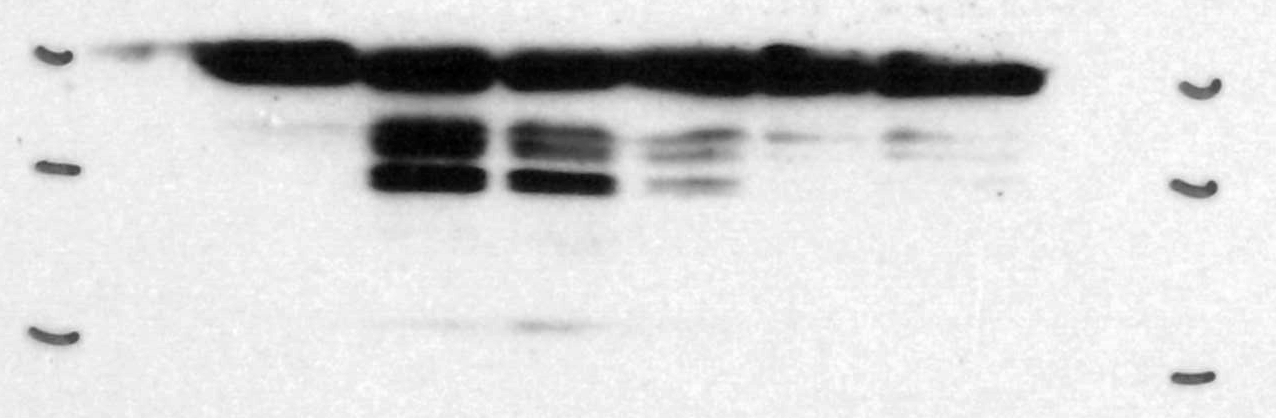

Supplement: Figure 1—source data 1. [file elife-89066-fig1-data1.zip › Figure 1 - source data 1/Figure 1a - source data/7. casp12.tiff]

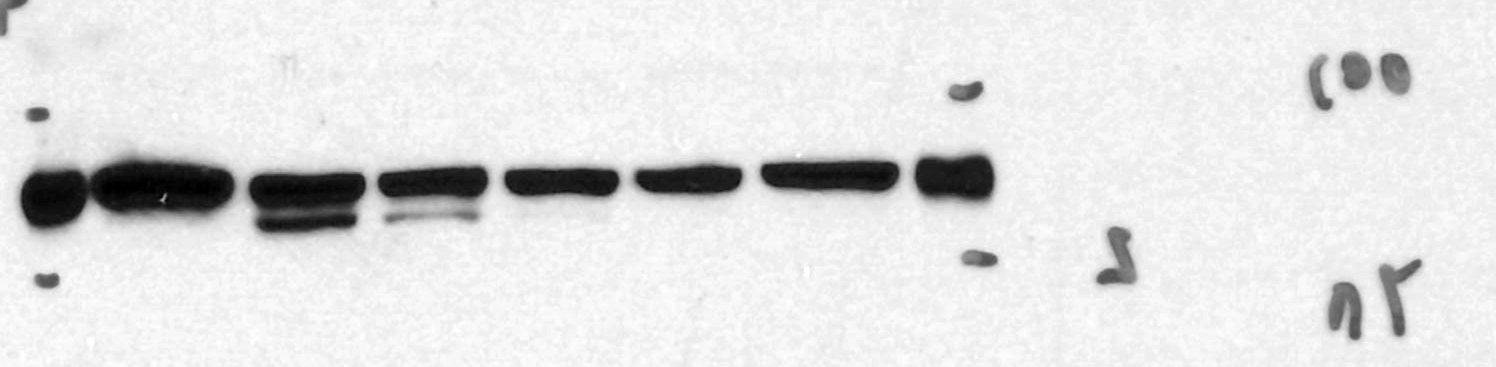

Supplement: Figure 1—source data 1. [file elife-89066-fig1-data1.zip › Figure 1 - source data 1/Figure 1a - source data/5. Nup93.tiff]

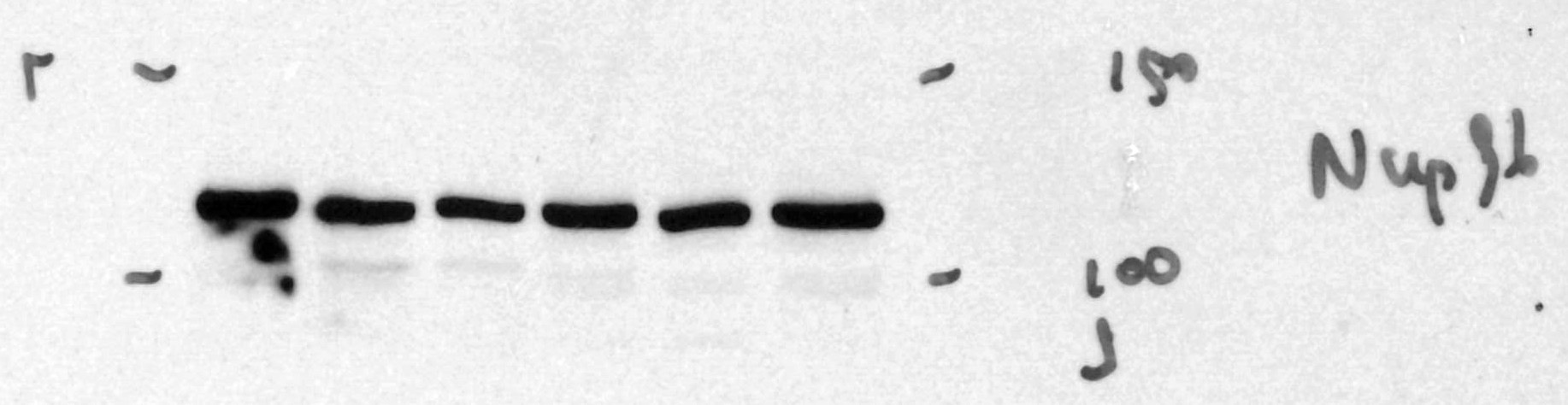

Supplement: Figure 1—source data 1. [file elife-89066-fig1-data1.zip › Figure 1 - source data 1/Figure 1a - source data/4. Nup96.tiff]

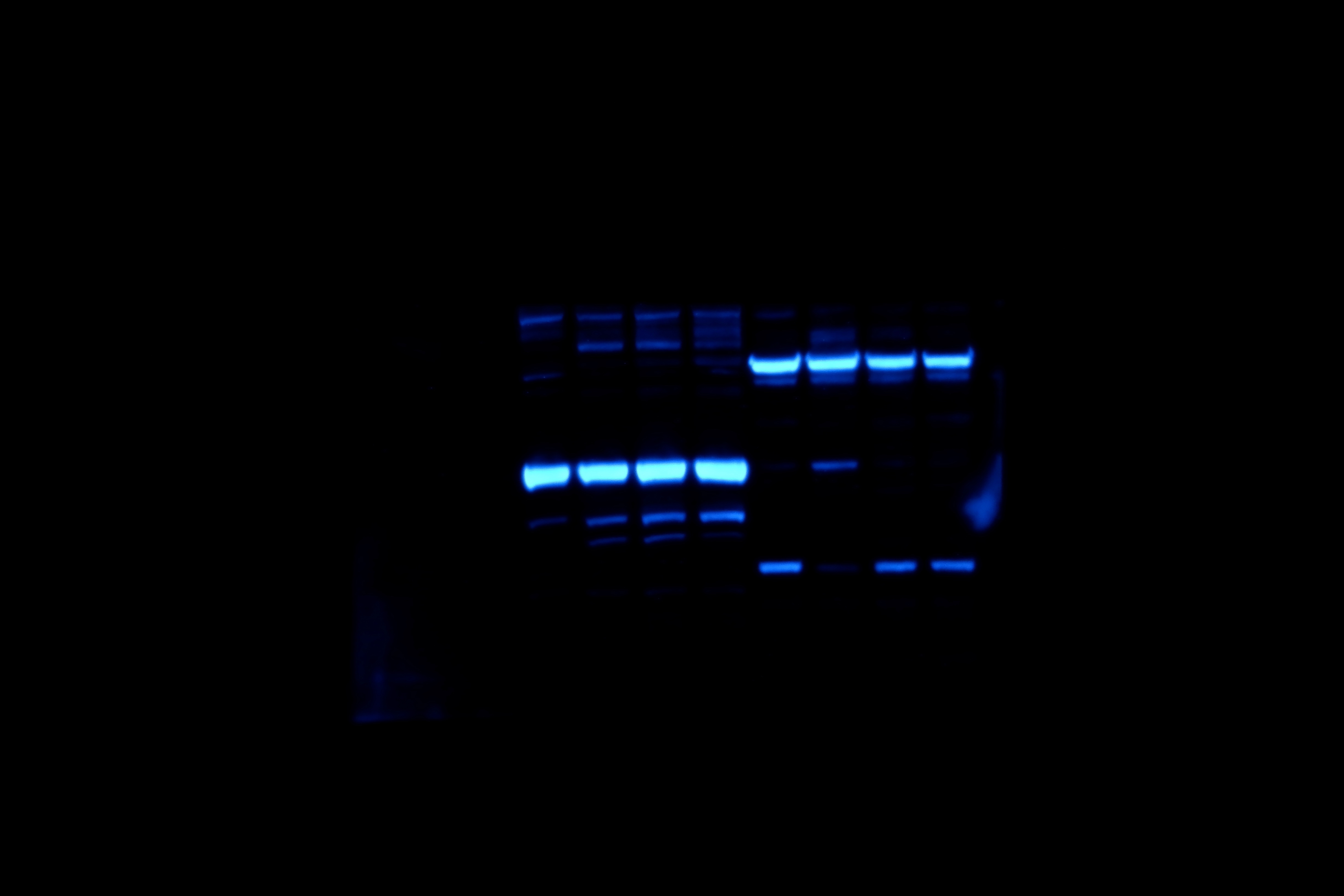

Supplement: Figure 1—source data 1. [file elife-89066-fig1-data1.zip › Figure 1 - source data 1/Figure 1f - source data/DSCF9940.JPG]

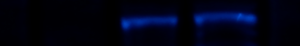

Supplement: Figure 1—source data 1. [file elife-89066-fig1-data1.zip › Figure 1 - source data 1/Figure 1b - source data/11. Nup37.tif]

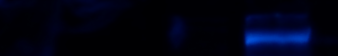

Supplement: Figure 1—source data 1. [file elife-89066-fig1-data1.zip › Figure 1 - source data 1/Figure 1b - source data/3. Pom121.tif]

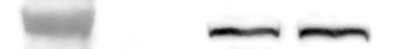

Supplement: Figure 1—source data 1. [file elife-89066-fig1-data1.zip › Figure 1 - source data 1/Figure 1b - source data/7. Nup160.tif]

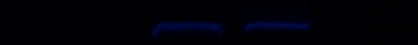

Supplement: Figure 1—source data 1. [file elife-89066-fig1-data1.zip › Figure 1 - source data 1/Figure 1b - source data/5 .Nup205.tif]

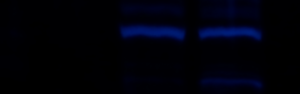

Supplement: Figure 1—source data 1. [file elife-89066-fig1-data1.zip › Figure 1 - source data 1/Figure 1b - source data/9. Nup85.tif]

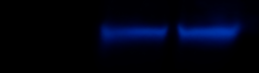

Supplement: Figure 1—source data 1. [file elife-89066-fig1-data1.zip › Figure 1 - source data 1/Figure 1b - source data/10. Sec13.tif]

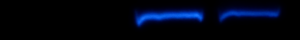

Supplement: Figure 1—source data 1. [file elife-89066-fig1-data1.zip › Figure 1 - source data 1/Figure 1b - source data/8. Nup155.tif]

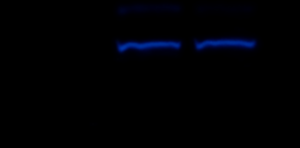

Supplement: Figure 1—source data 1. [file elife-89066-fig1-data1.zip › Figure 1 - source data 1/Figure 1b - source data/1. lamin b1.tif]

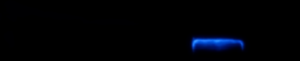

Supplement: Figure 1—source data 1. [file elife-89066-fig1-data1.zip › Figure 1 - source data 1/Figure 1b - source data/2. Tpr.tif]

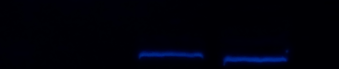

Supplement: Figure 1—source data 1. [file elife-89066-fig1-data1.zip › Figure 1 - source data 1/Figure 1b - source data/4. ELYS.tif]

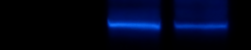

Supplement: Figure 1—source data 1. [file elife-89066-fig1-data1.zip › Figure 1 - source data 1/Figure 1b - source data/6. Nup188.tif]

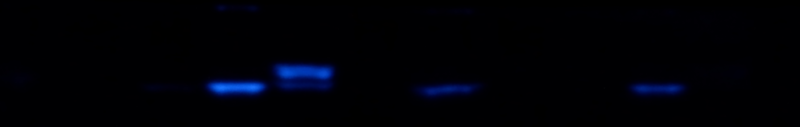

Supplement: Figure 2—source data 1. [file elife-89066-fig2-data1.zip › Figure 2 - source data 1/3. Caspase-3.tif]

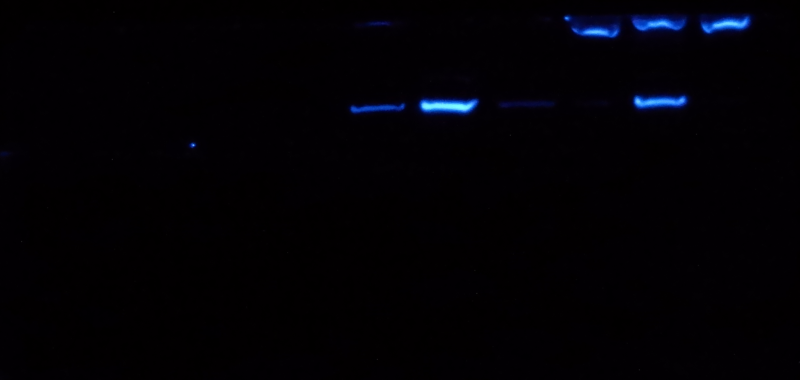

Supplement: Figure 2—source data 1. [file elife-89066-fig2-data1.zip › Figure 2 - source data 1/2. Tpr.tif]

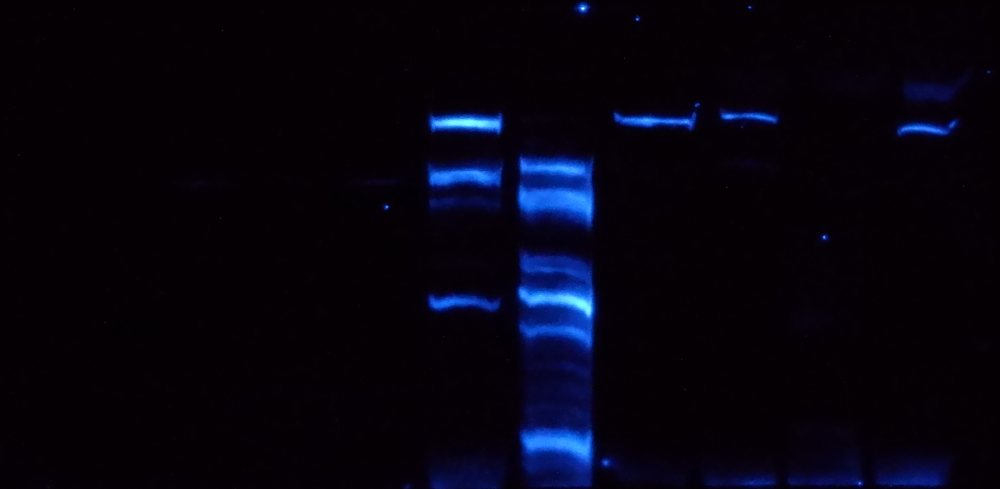

Supplement: Figure 2—source data 1. [file elife-89066-fig2-data1.zip › Figure 2 - source data 1/1. Nup153.tif]

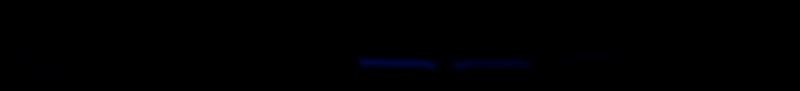

Supplement: Figure 3—source data 1. [file elife-89066-fig3-data1.zip › Figure 3 - source data 1/5. Nup93.tif]

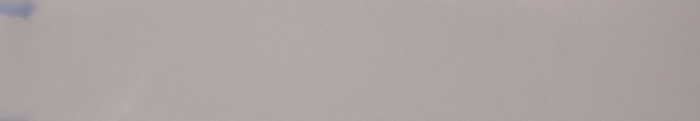

Supplement: Figure 3—source data 1. [file elife-89066-fig3-data1.zip › Figure 3 - source data 1/12. Myogenin.tif]

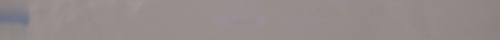

Supplement: Figure 3—source data 1. [file elife-89066-fig3-data1.zip › Figure 3 - source data 1/6. Pom121.tif]

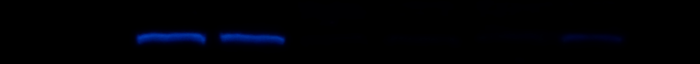

Supplement: Figure 3—source data 1. [file elife-89066-fig3-data1.zip › Figure 3 - source data 1/8. FAK.tif]

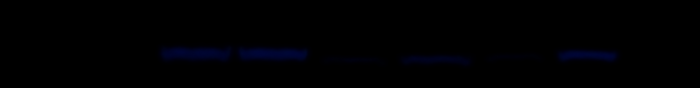

Supplement: Figure 3—source data 1. [file elife-89066-fig3-data1.zip › Figure 3 - source data 1/11. a-tubulin.tif]

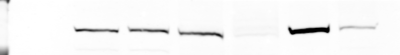

Supplement: Figure 3—source data 1. [file elife-89066-fig3-data1.zip › Figure 3 - source data 1/7. PARP.tif]

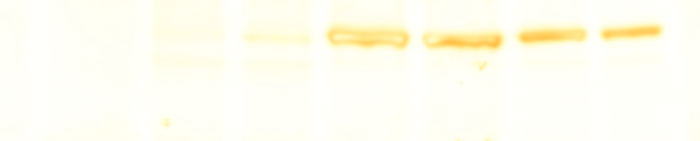

Supplement: Figure 3—source data 1. [file elife-89066-fig3-data1.zip › Figure 3 - source data 1/2. Fibrillarin.tif]

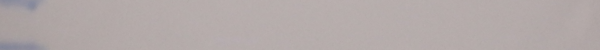

Supplement: Figure 3—source data 1. [file elife-89066-fig3-data1.zip › Figure 3 - source data 1/13. Caspase-3.tif]

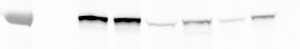

Supplement: Figure 3—source data 1. [file elife-89066-fig3-data1.zip › Figure 3 - source data 1/10. Hic-5.tif]

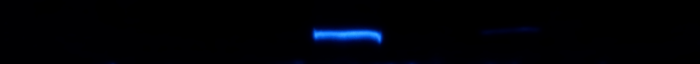

Supplement: Figure 3—source data 1. [file elife-89066-fig3-data1.zip › Figure 3 - source data 1/4. Nup153.tif]

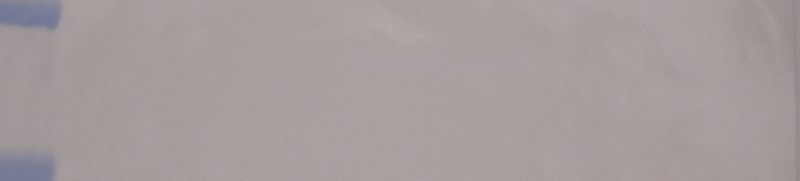

Supplement: Figure 3—source data 1. [file elife-89066-fig3-data1.zip › Figure 3 - source data 1/1. Lamin b1.tif]

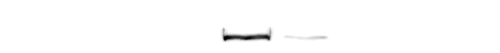

Supplement: Figure 3—source data 1. [file elife-89066-fig3-data1.zip › Figure 3 - source data 1/3. Tpr.tif]

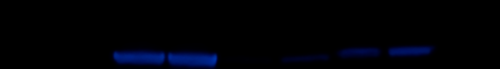

Supplement: Figure 3—source data 1. [file elife-89066-fig3-data1.zip › Figure 3 - source data 1/9. Zyxin.tif]

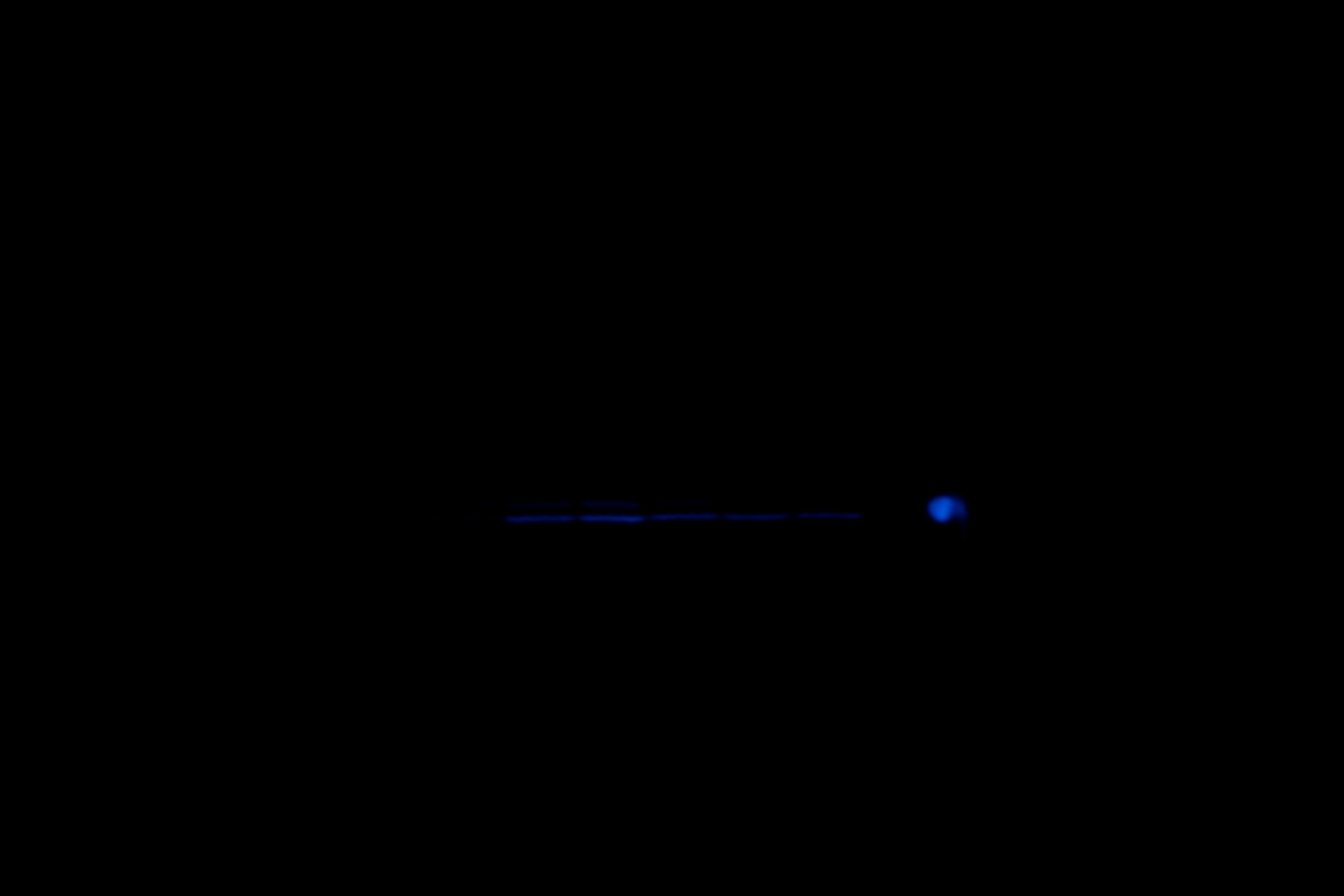

Supplement: Figure 5—source data 1. [file elife-89066-fig5-data1.zip › Figure 5 - source data 1/Figure 5d - source data/3. Caspase-3.JPG]

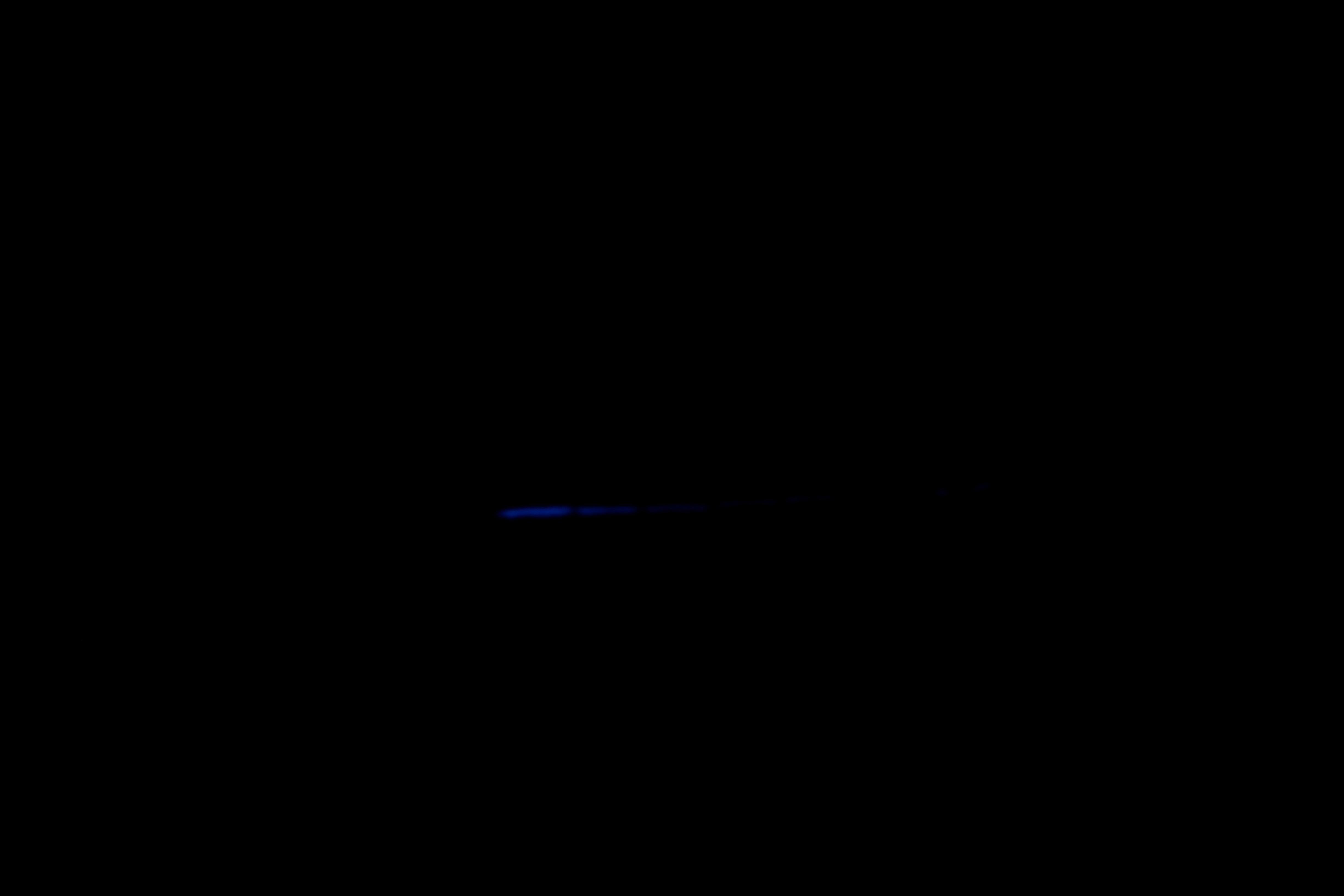

Supplement: Figure 5—source data 1. [file elife-89066-fig5-data1.zip › Figure 5 - source data 1/Figure 5d - source data/4. Survivin.JPG]

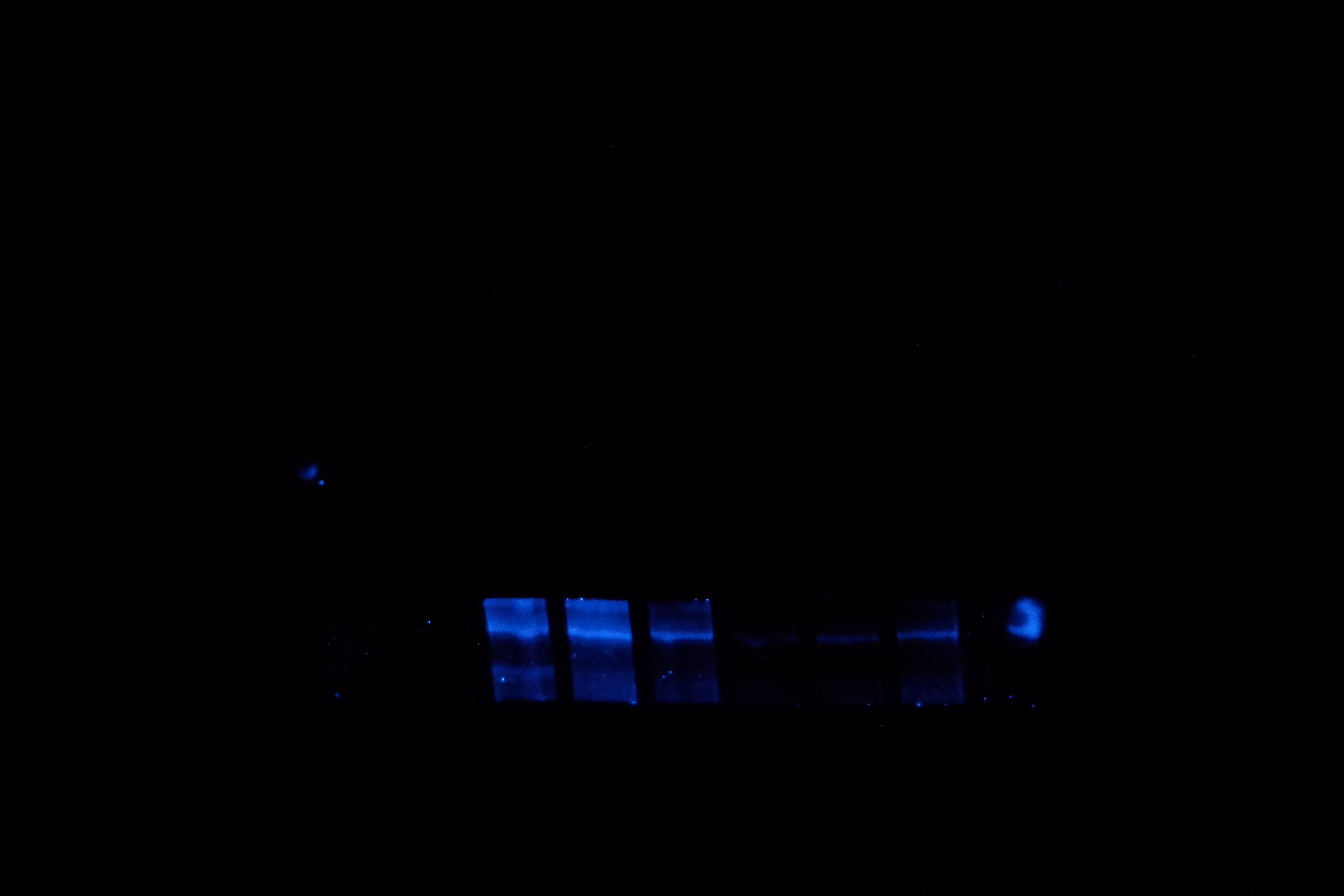

Supplement: Figure 5—source data 1. [file elife-89066-fig5-data1.zip › Figure 5 - source data 1/Figure 5d - source data/1. Nup153.JPG]

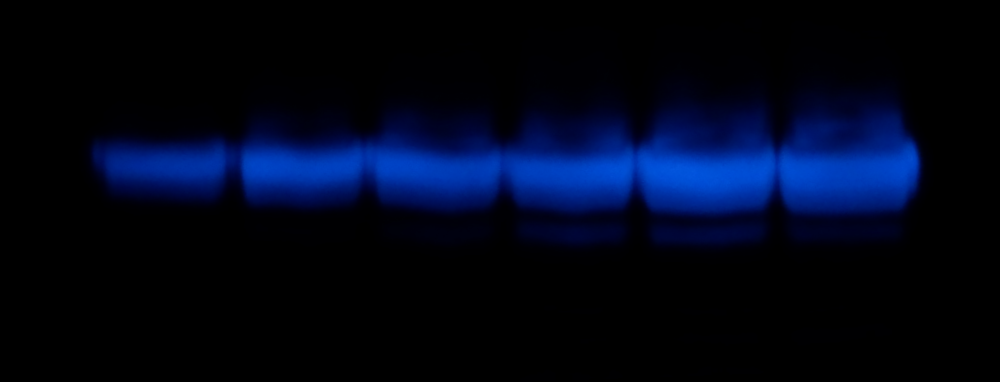

Supplement: Figure 5—source data 1. [file elife-89066-fig5-data1.zip › Figure 5 - source data 1/Figure 5d - source data/6. Tubulin bIII.tif]

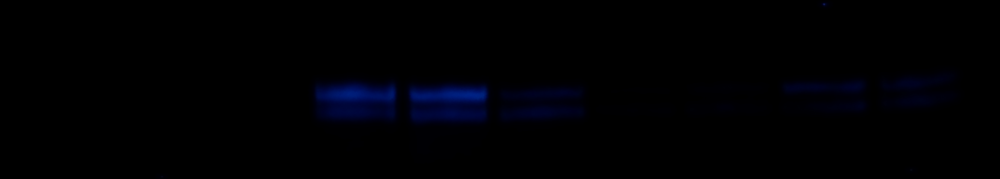

Supplement: Figure 5—source data 1. [file elife-89066-fig5-data1.zip › Figure 5 - source data 1/Figure 5d - source data/5. Sox2.tif]

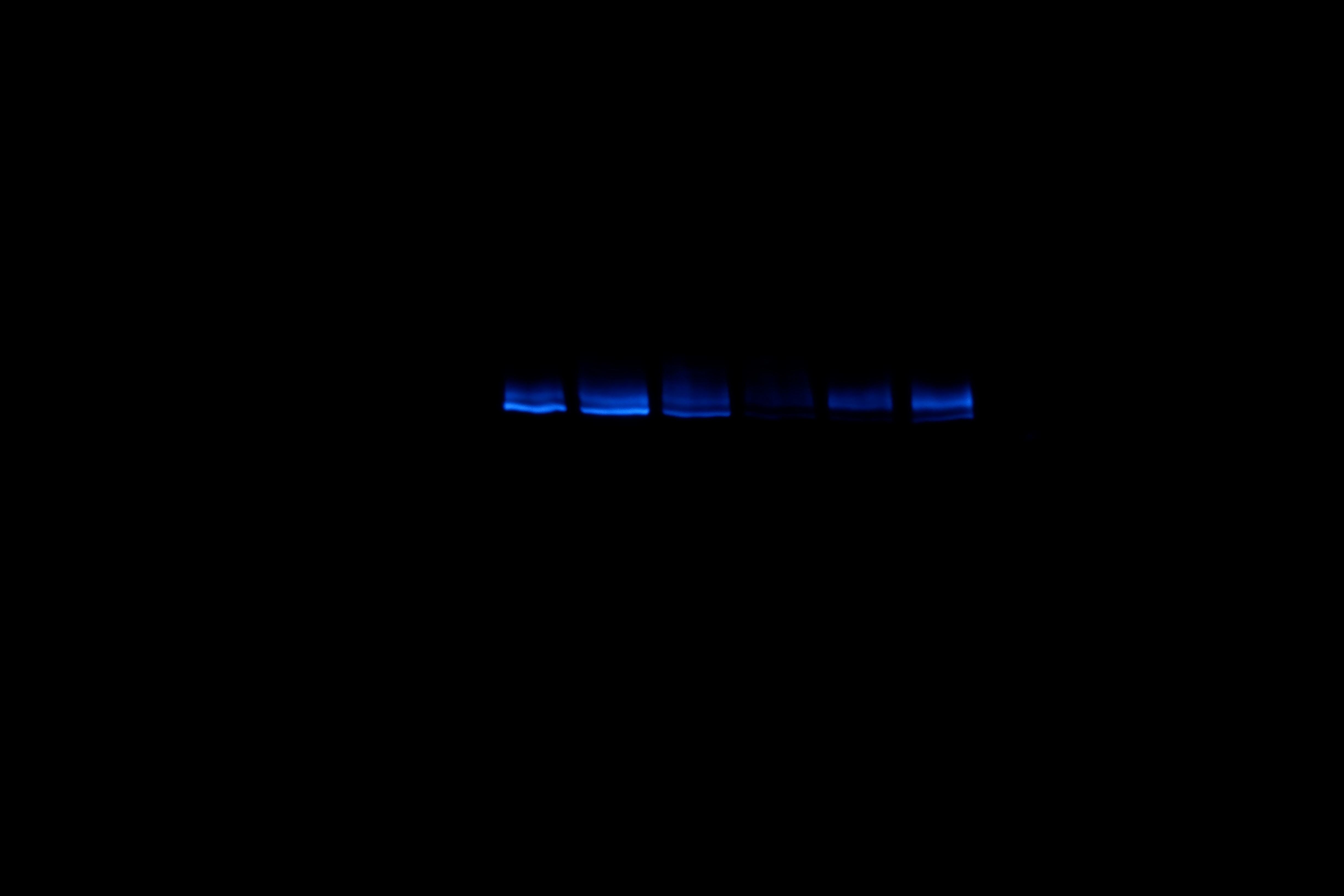

Supplement: Figure 5—source data 1. [file elife-89066-fig5-data1.zip › Figure 5 - source data 1/Figure 5d - source data/2. PARP.JPG]

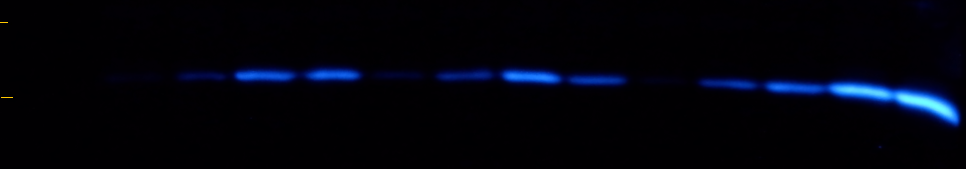

Supplement: Figure 5—source data 1. [file elife-89066-fig5-data1.zip › Figure 5 - source data 1/Figure 5e - source data/8. Caspase-3.tif]

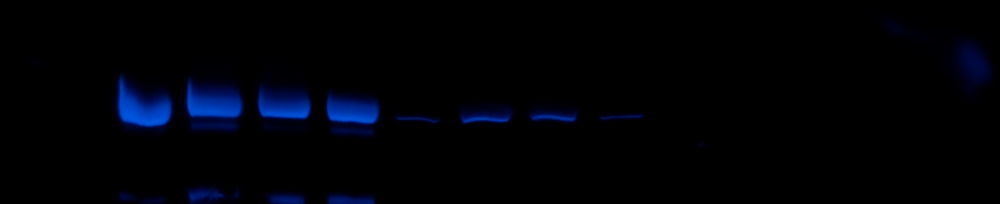

Supplement: Figure 5—source data 1. [file elife-89066-fig5-data1.zip › Figure 5 - source data 1/Figure 5e - source data/6. MHC.tif]

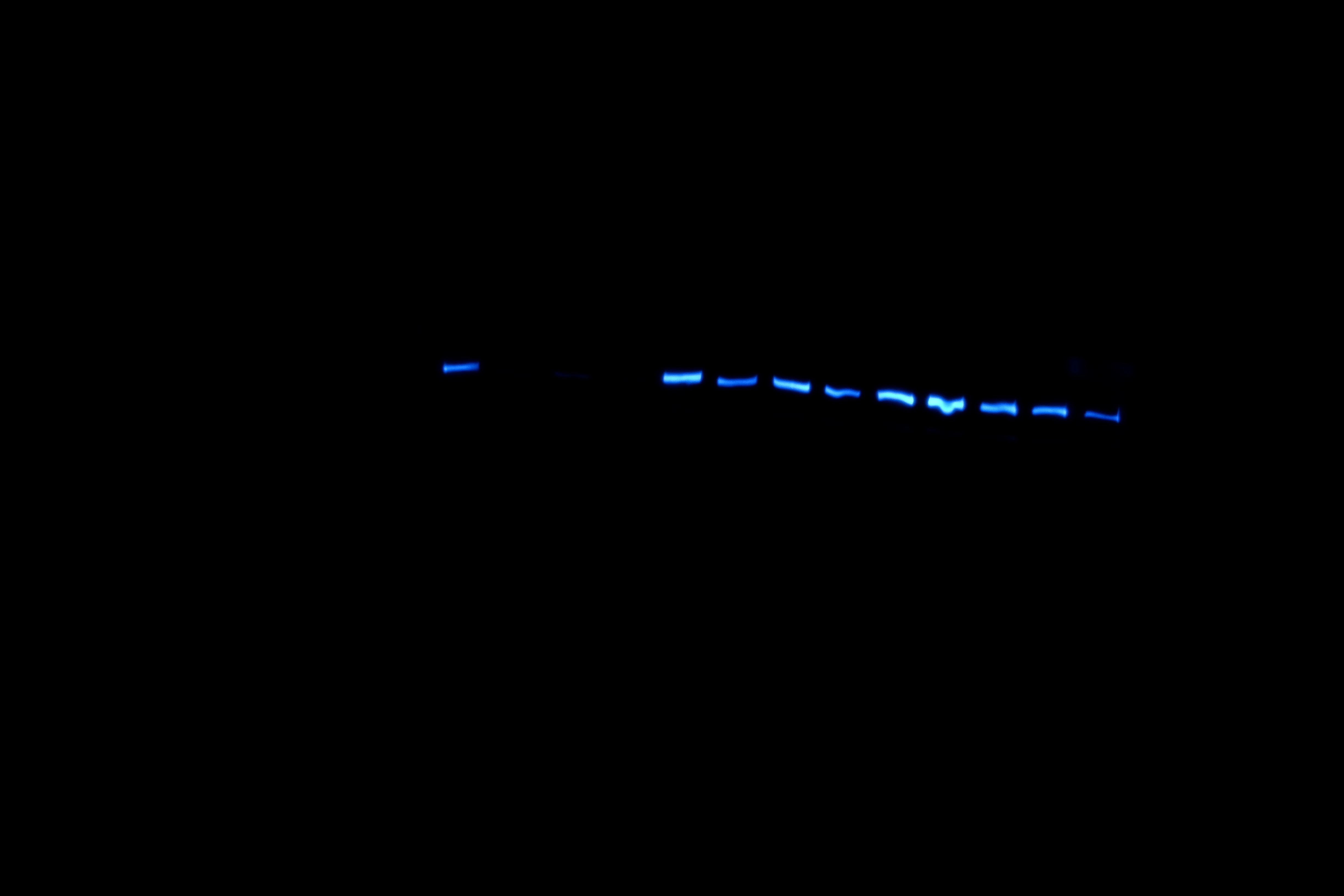

Supplement: Figure 5—source data 1. [file elife-89066-fig5-data1.zip › Figure 5 - source data 1/Figure 5e - source data/1. Tpr.JPG]

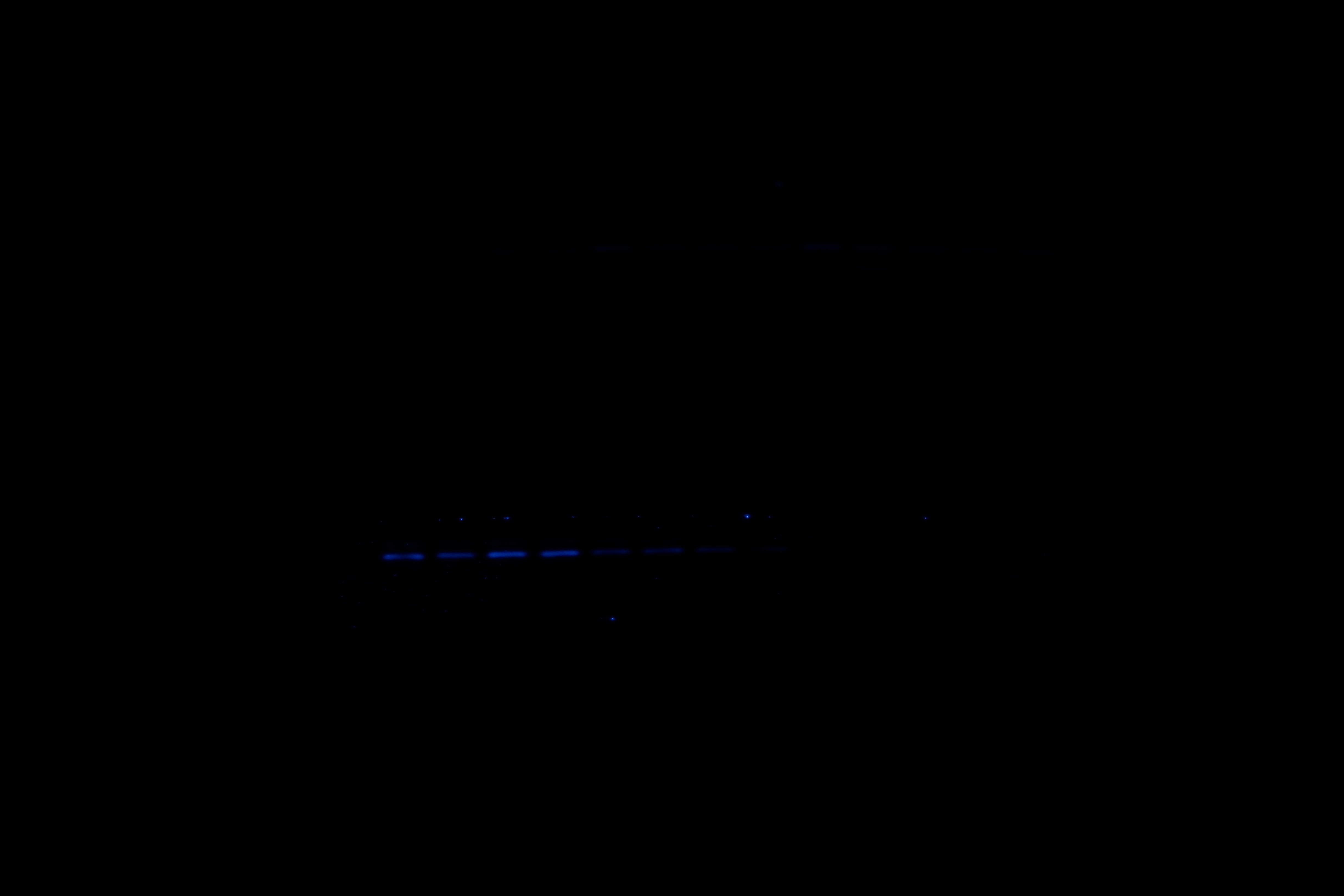

Supplement: Figure 5—source data 1. [file elife-89066-fig5-data1.zip › Figure 5 - source data 1/Figure 5e - source data/7. Myogenin.JPG]

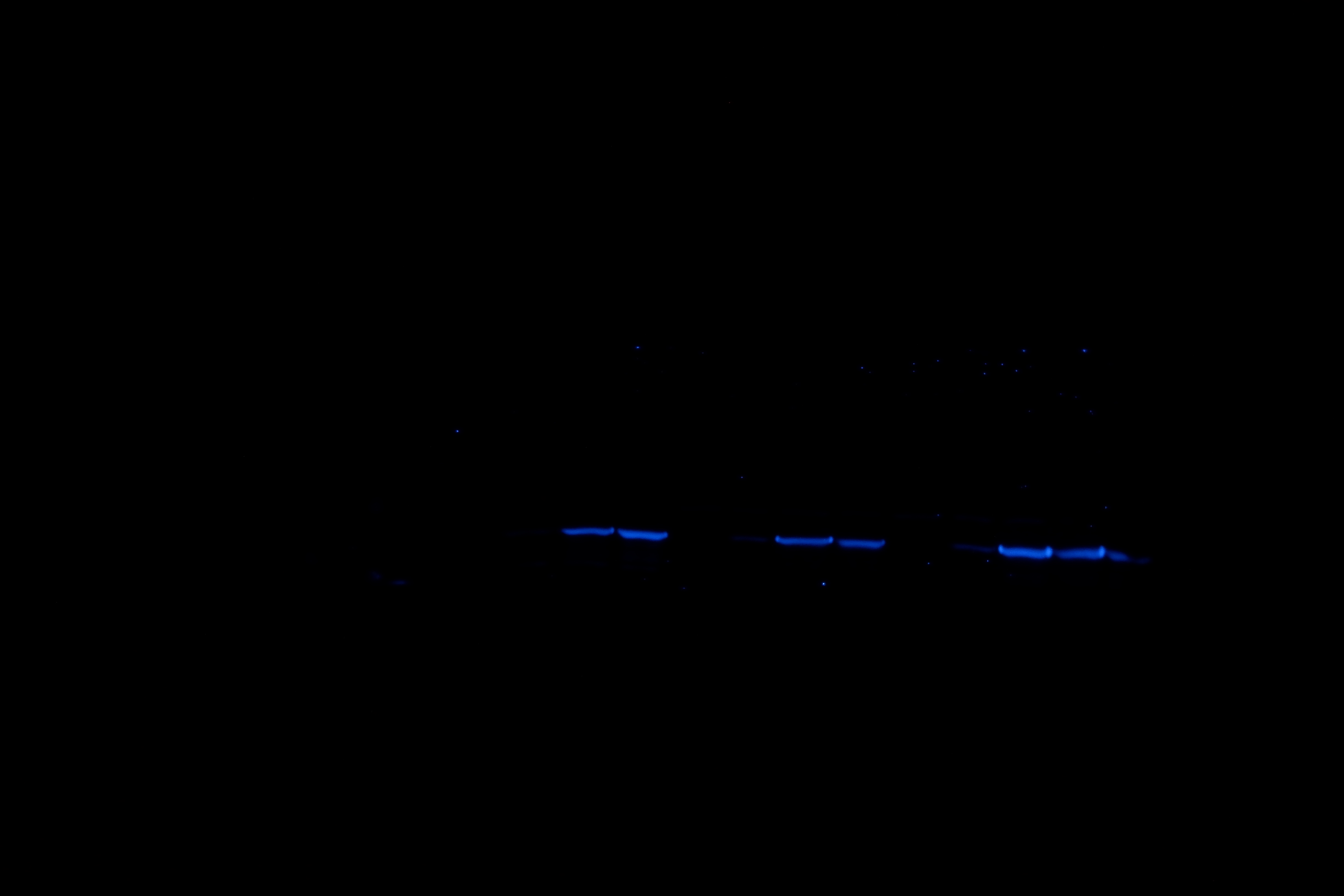

Supplement: Figure 5—source data 1. [file elife-89066-fig5-data1.zip › Figure 5 - source data 1/Figure 5e - source data/4. BiP.JPG]

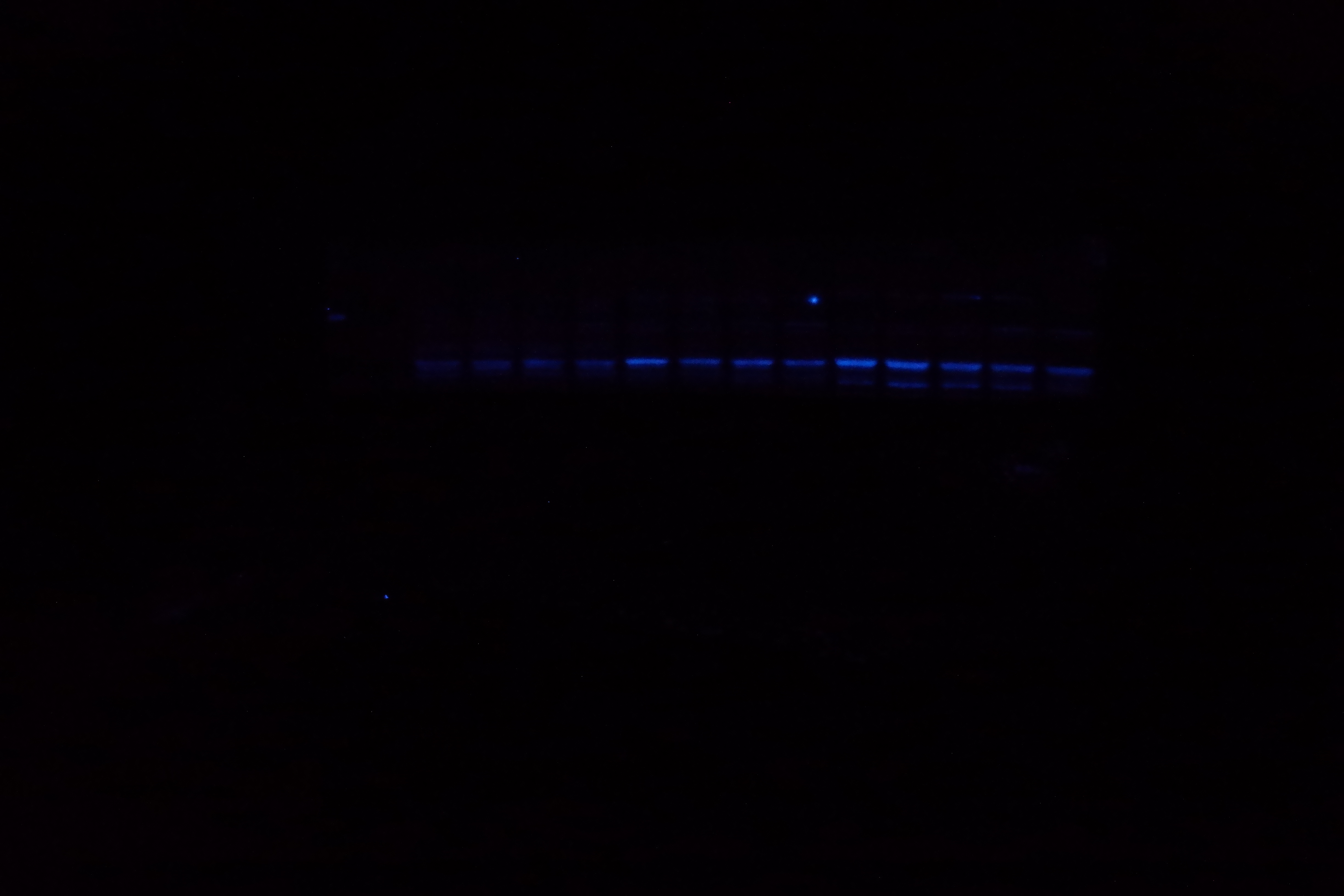

Supplement: Figure 5—source data 1. [file elife-89066-fig5-data1.zip › Figure 5 - source data 1/Figure 5e - source data/5. XIAP.JPG]

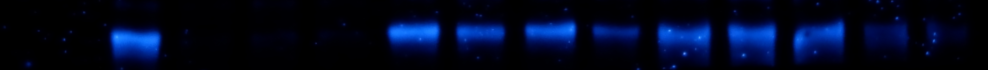

Supplement: Figure 5—source data 1. [file elife-89066-fig5-data1.zip › Figure 5 - source data 1/Figure 5e - source data/2. Nup153.tif]

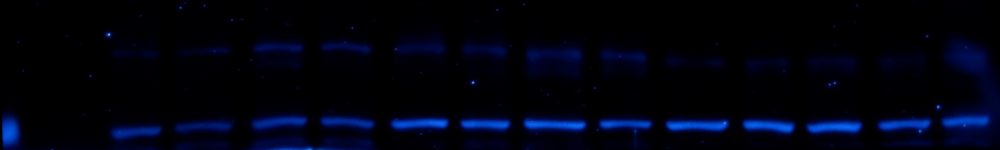

Supplement: Figure 5—source data 1. [file elife-89066-fig5-data1.zip › Figure 5 - source data 1/Figure 5e - source data/3. Nup93.tif]

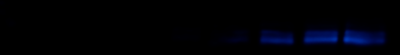

Supplement: Figure 5—source data 1. [file elife-89066-fig5-data1.zip › Figure 5 - source data 1/Figure 5b - source data/3. Myogenin.tif]

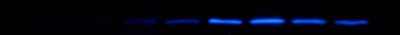

Supplement: Figure 5—source data 1. [file elife-89066-fig5-data1.zip › Figure 5 - source data 1/Figure 5b - source data/2. Caspase-3.tif]

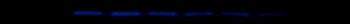

Supplement: Figure 5—source data 1. [file elife-89066-fig5-data1.zip › Figure 5 - source data 1/Figure 5b - source data/1. Survivin.tif]

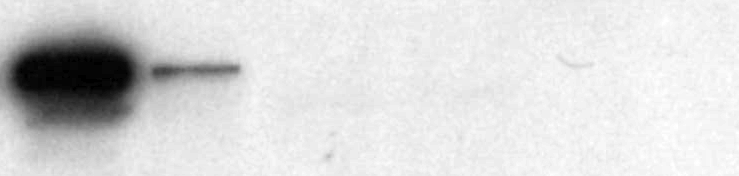

Supplement: Figure 5—source data 1. [file elife-89066-fig5-data1.zip › Figure 5 - source data 1/Figure 5f - source data/2. Tpr.tiff]

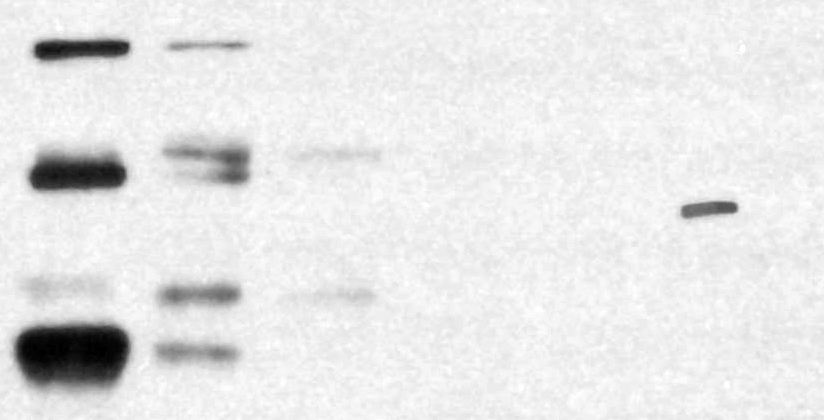

Supplement: Figure 5—source data 1. [file elife-89066-fig5-data1.zip › Figure 5 - source data 1/Figure 5f - source data/1. Nup358 214 153.tiff]

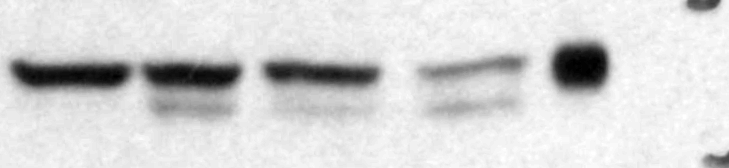

Supplement: Figure 5—source data 1. [file elife-89066-fig5-data1.zip › Figure 5 - source data 1/Figure 5f - source data/3. Nup93.tiff]

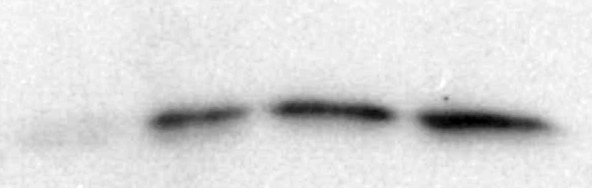

Supplement: Figure 5—source data 1. [file elife-89066-fig5-data1.zip › Figure 5 - source data 1/Figure 5f - source data/5. Caspase-3.tiff]

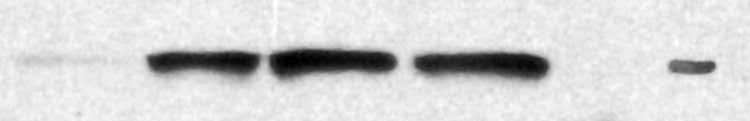

Supplement: Figure 5—source data 1. [file elife-89066-fig5-data1.zip › Figure 5 - source data 1/Figure 5f - source data/4. BiP.tiff]
