# Supplementary material for: Caspase-mediated nuclear pore complex trimming in cell differentiation and endoplasmic reticulum stress
Source: eLife. 2023 Sep 4;12:RP89066. doi: 10.7554/eLife.89066 (PMC10476967; doi:10.7554/eLife.89066)
Supplement: Supplementary file 2. — (a) Primary antibodies used in this study (b) Secondary antibodies used in this study (c) Plasmids used in this study (d) RNA FISH probes used in the study (e) Chemicals used in this study [file elife-89066-supp2.docx]

**Supplementary File 2a. Primary antibodies used in this study**

| **Antigen group** | **Antigen** | **Host** | **Clonality** | **Application** | **Dilution** | **Source** |
| --- | --- | --- | --- | --- | --- | --- |
| Nups | Tpr | Rabbit | Polyclonal | Western blot | 1:1000 | Abcam (ab84516) |
|  |  |  |  | Immunofluorescence | 1:300 |  |
|  |  | Mouse | Monoclonal | Immunofluorescence | 1:200 | Santa Cruz (sc-271565) |
|  | Nup358  Nup214  Nup153  Nup62 | Mouse | Monoclonal | Western blot | 1:4000 | Covance (MMS-120R) |
|  |  |  |  | Immunofluorescence | 1:500 |  |
|  | Nup153 | Mouse | Monoclonal | Immunofluorescence | 1:3 | In-house (SA1) |
|  |  | Rat | Monoclonal | Immunofluorescence | 1:300 | Abcam (ab81463) |
|  | Nup96 | Rabbit | Monoclonal | Western blot | 1:1000 | Abcam (ab124980) |
|  | Nup93 | Mouse | Monoclonal | Western blot | 1:200 | Santa Cruz (sc-81343) |
|  | Nup50 | Rabbit | Monoclonal | Western blot | 1:1000 | Abcam (ab151567) |
|  | ELYS | Rabbit | Polyclonal | Western blot | 1:1000 | In-house |
|  | Nup205 | Rabbit | Polyclonal | Western blot | 1:500 | In-house |
|  | Nup188 | Rabbit | Polyclonal | Western blot | 1:1000 | Bethyl Laboratories (A302-323A) |
|  | Nup160 | Rabbit | Polyclonal | Western blot | 1:500 | In-house |
|  | Nup155 | Guinea Pig | Polyclonal | Western blot | 1:1000 | In-house |
|  | Nup85 | Rabbit | Polyclonal | Western blot | 1:500 | In-house |
|  | Sec13 | Rabbit | Polyclonal | Western blot | 1:500 | In-house |
|  | Nup37 | Rabbit | Polyclonal | Western blot | 1:500 | In-house |
|  | Pom121 | Rabbit | Polyclonal | Western blot | 1:1000 | Invitrogen (PA5-36498) |
|  |  |  |  | Immunofluorescence | 1:300 |  |
| Caspases | Caspase-3 | Rabbit | Polyclonal | Western blot | 1:1000 | Cell Signaling Technology (9661) |
|  | Caspase-9 | Rabbit | Polyclonal | Western blot | 1:1000 | Cell Signaling Technology (9504) |
|  | Caspase-12 | Rat | Monoclonal | Western blot | 1:200 | Santa Cruz (sc-21747) |
| IAPs | XIAP | Rabbit | Polyclonal | Western blot | 1:1000 | Cell Signaling Technology (2042) |
|  | Survivin | Rabbit | Monoclonal | Western blot | 1:1000 | Cell Signaling Technology (2808) |
| Differentiation-  related | Cleaved Notch1 | Rabbit | Monoclonal | Western blot | 1:1000 | Cell Signaling Technology (4147) |
|  | Myogenin | Mouse | Monoclonal | Western blot | 1:1000 | BD Biosciences (556358) |
|  |  |  |  | Immunofluorescence | 1:200 |  |
|  | Myosin heavy chain | Mouse | Monoclonal | Western blot | 1:100 | In-house (MF20) |
|  | Sox2 | Rabbit | Polyclonal | Western blot | 1:1000 | Cell Signaling Technology (2748) |
|  | βIII-Tubulin | Rabbit | Polyclonal | Western blot | 1:5000 | Biolegend (802001) |
| ER stress | Bip | Rabbit | Monoclonal | Western blot | 1:1000 | Cell Signaling Technology (3177) |
| Caspase  substrates | PARP | Rabbit | Monoclonal | Western blot | 1:1000 | Cell Signaling Technology (9532) |
|  | αII-Spectrin | Mouse | Monoclonal | Western blot | 1:100 | Santa Cruz (sc-48382) |
| Cytoplasm &  nucleoplasm  markers | α-Tubulin | Mouse | Monoclonal | Western blot | 1:5000 | Sigma (T5168) |
|  | Lamin B1 | Mouse | Monoclonal | Western blot | 1:200 | Santa Cruz (sc-374015) |
|  | Fibrillarin | Chicken | Polyclonal | Western blot | 1:2000 | Novus Biologicals (NBP2-46881) |
| Focal adhesion  proteins | Hic-5 | Rabbit | Polyclonal | Western blot | 1:1000 | Proteintech (10565-1-AP) |
|  | Zyxin | Mouse | Monoclonal | Western blot | 1:1000 | R&D Systems (MAB6977) |
|  | Paxillin | Mouse | Monoclonal | Western blot | 1:1000 | Invitrogen (AHO0492) |
|  | FAK | Mouse | Monoclonal | Western blot | 1:1000 | BD Biosciences (610087) |
|  |  | Rabbit | Polyclonal | Western blot | 1:1000 | Cell Signaling Technology (3285) |
| Karyopherins | Crm1 | Mouse | Monoclonal | Immunofluorescence | 1:200 | BD Biosciences (611833) |
|  | Importin-α | Mouse | Monoclonal | Immunofluorescence | 1:1000 | Abcam (ab2811) |
|  | Importin-β | Rabbit | Polycloncal | Immunofluorescence | 1:200 | Novus Biologicals (NBP2-38482) |
| RNA polymerase | Phospho-Rbp1 (Ser5) | Rabbit | Monoclonal | Western blot | 1:1000 | Cell Signaling Technology (13523) |

**Supplementary File 2b. Secondary antibodies used in this study**

| **Antibody** | **Host** | **Clonality** | **Application** | **Dilution** | **Source** |
| --- | --- | --- | --- | --- | --- |
| HRP-conjugated anti-mouse IgG | Goat | Polyclonal | Western blot | 1:10,000 | Invitrogen (G-21040) |
| HRP-conjugated anti-rabbit IgG | Goat |  |  | 1:10,000 | Invitrogen (G-21234) |
| HRP-conjugated anti-rat IgG | Goat |  |  | 1:3,000 | Invitrogen (A10549) |
| HRP-conjugated anti-chicken IgY | Goat |  |  | 1:3000 | Invitrogen (A16054) |
| HRP-conjugated anti-guinea pig IgG | Goal |  |  | 1:3000 | Invitrogen (A18775) |
| IRDye800-conjugated  anti-mouse IgG | Donkey |  |  | 1:10,000 | Rockland Immunochemicals  (610-732-124) |
| Alexa Fluor 680-conjugated  Anti-rabbit IgG | Goat |  |  | 1:10,000 | Invitrogen (A-21109) |
| Alexa Fluor 647-conjugated  anti-mouse IgG | Donkey |  | Immunofluorescence | 1:1000 | Invitrogen (A-31571) |
| Alexa Fluor 568-conjugated  Anti-rabbit IgG | Goat |  |  | 1:1000 | Invitrogen (A-11036) |
| Alexa Fluor 488-conjugated  anti-rat IgG | Goat |  |  | 1:1000 | Invitrogen (A-11006) |

**Supplementary File 2c. Plasmids used in this study**

| **No.** | **Plasmid** | **Backbone** | **Insert** | **Insert PCR template** | **Insert PCR Primers (5’ to 3’)** | **Cloning method** |
| --- | --- | --- | --- | --- | --- | --- |
| 1 | Doxycycline-inducible  GFP-myogenin | LT3GEPIR  (Addgene  111177) | Myogenin | GE Healthcare  Dharmacon, Inc.  MMM1013-202805422 | Forward: atcgtgtacaagtcatcagagctgtatgagacatccccctatttc  Reverse: atcggaattctcagttgggcatggtttcgtc | Backbone and insert cut with BsrG1 and EcoR1 were ligated  by T7 DNA ligase. |
| 2 | Doxycycline-inducible  GFP-myogenin |  | GFP-myogenin | Plasmid No. 1 | Forward: aagtcgagcttgcgttggatc  Reverse: aaggcacagtgtacatcagttgggcatggtttcgtc | PCR products were cloned into the backbone cut with BamH1 and EcoR1  by In-fusion cloning. |
|  |  |  | Bovine growth hormone Poly-A | N/A | Forward: actgatgtacactgtgccttctagttgcc  Reverse: acaagataattgctcgaattcccatagagcccaccgcatc |  |
| 3 | EF1α promoter  NES-eGFP | pEF1α-Tet3G | NES_Rev_-GFP  -IRES-Puro^r^ | Plasmid DNA and map available upon request | | |
| 4 |  |  | NES_PKI_-GFP  -IRES-Puro^r^ |  |  |  |

NES_Rev_: LPPLERLTL

NES_PKI_: LALKLAGLDI

**Supplementary File 2d. RNA FISH probes used in the study**

| **RNA target** | **Label** | **Source** | **Probe type** | **Sequence used to generate**  **Stellaris custom probe sets**  (NCBI reference sequence) |
| --- | --- | --- | --- | --- |
| 18S rRNA | Quasar 570 | Stellaris | Deoxyribonucleic acid | NR_003278.3^1^ |
| *Gapdh* mRNA | Quasar 670 |  |  | NM_008084.2 (ShipReady, SMF-3140-1) |
| Poly-A RNA | TYE563 | Qiagen | Locked nucleic acid (T_25_) |  |

**Supplementary File 2e. Chemicals used in this study**

| **Chemical** | **Function** | **Stock solution solvent** | **Stock solution concentration** | **Working concentration** | **Source** |
| --- | --- | --- | --- | --- | --- |
| Q-VD(OMe)-OPh | Pan-caspase inhibitor | DMSO | 30 mM | 30 μM | APExBIO Technology (A8165) |
| Z-LL-CHO | Pan-calpain inhibitor |  | 50 mM | 50 μM | Peptide Institute, Inc. (IZL-3178-v) |
| FK506 | Calcineurin inhibitor |  | 1 mg/ml | 0-100 ng/ml | Enzo Life Sciences (ALX-380-008) |
| DAPT | γ-Secretase inhibitor |  | 5 mM | 0-10 μM | Enzo Life Sciences (ALX-270-416) |
| Tunicamycin | ER stress inducer |  | 10 mg/ml | 0-1 μg/ml | Tocris (3516) |
| SCH772984 | ERK1/2 inhibitor |  | 10 mM | 1 μM | BioVision (B1682-5) |
| Doxycycline | Tetracycline transactivator activator | H_2_O | 1 mg/ml | 0-1000 ng/ml | Alfa Aesar (J60422) |
| Leptomycin B | Exportin-1 inhibitor | ethanol | 250 μM | 0-25 nM | BioVision (1814) |

**References**

1 Moor, A. E. *et al.* Global mRNA polarization regulates translation efficiency in the intestinal epithelium. *Science* **357**, 1299-1303 (2017).
