# Supplementary material for: Caspase-mediated nuclear pore complex trimming in cell differentiation and endoplasmic reticulum stress
Source: eLife. 2023 Sep 4;12:RP89066. doi: 10.7554/eLife.89066 (PMC10476967; doi:10.7554/eLife.89066)

Ponceau S staining – Related to Figures 1, 2, 3, and 5

Staining levels were determined by densitometric analysis. Normalized against lanes marked with arrowheads.

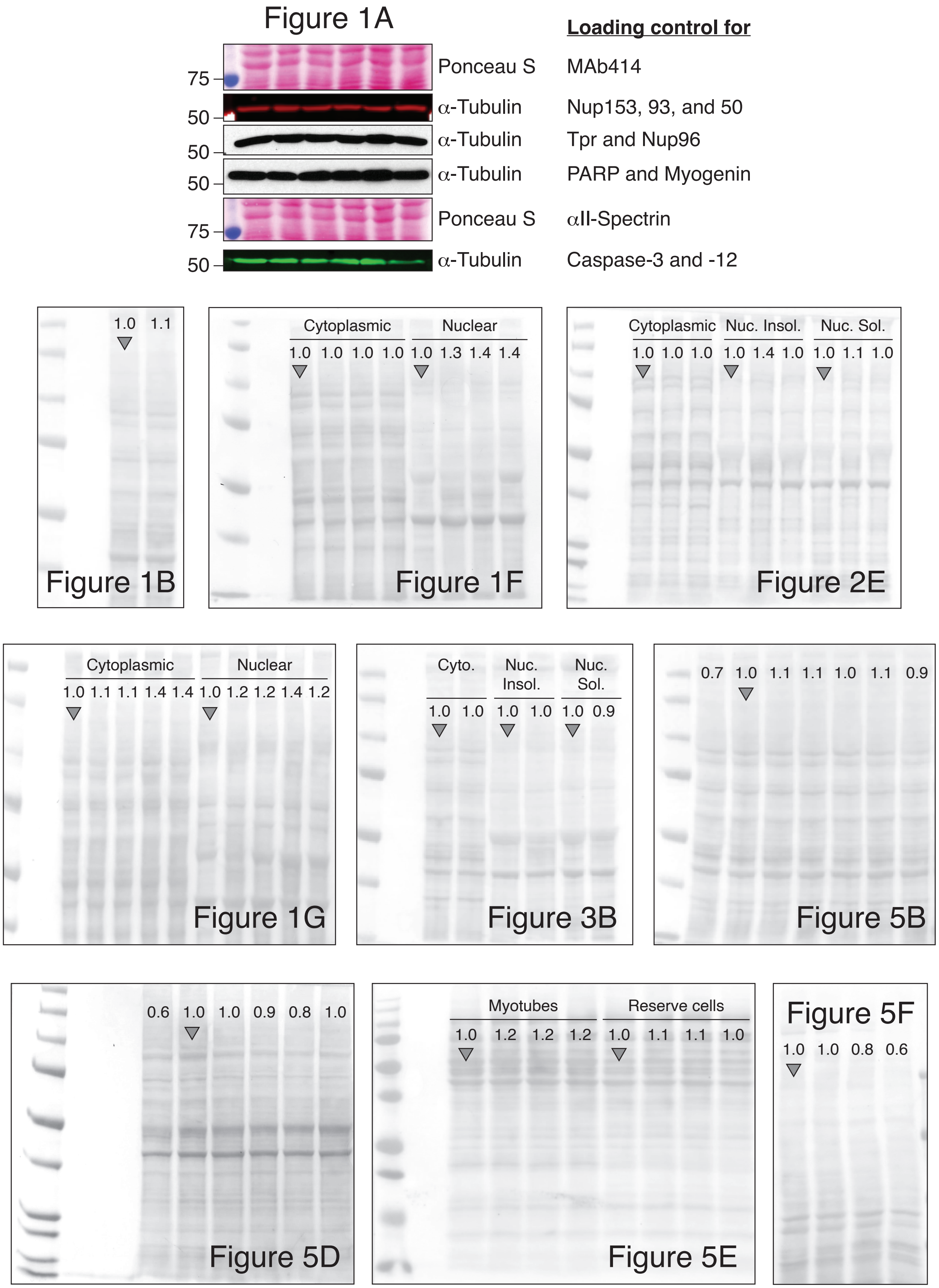

Ponceau S staining – Related to Supplementary Figures

Staining levels were determined by densitometric analysis. Normalized against lanes marked with arrowheads.

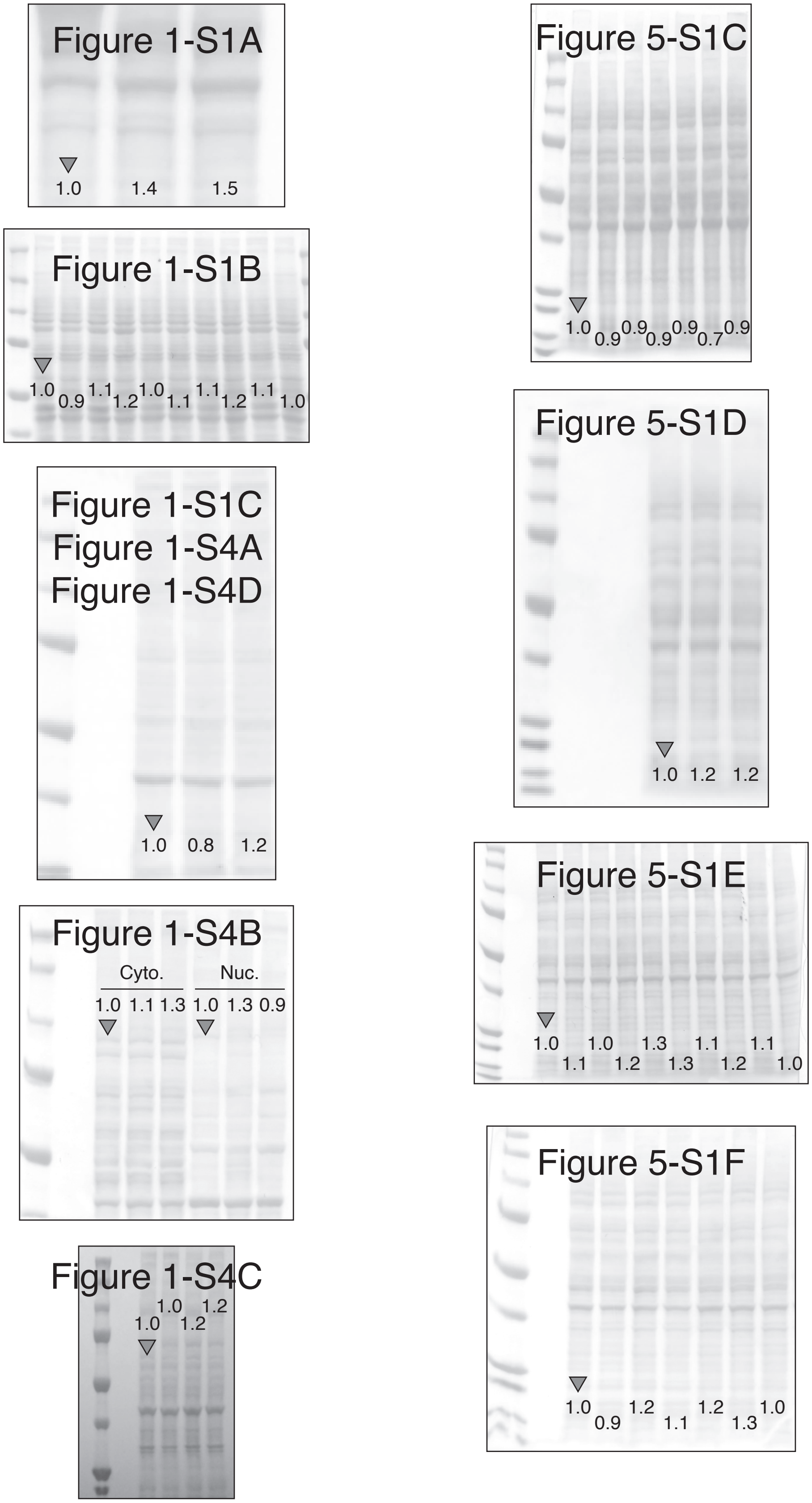

Supplement: Source data 1. [file elife-89066-data1.pdf]
